# Supplementary material for: The epidemiology and evolution of IgA nephropathy over two decades: A single centre experience
Source: PLoS One. 2022 Sep 1;17(9):e0268421. doi: 10.1371/journal.pone.0268421 (PMC9436111; doi:10.1371/journal.pone.0268421)
Supplement: S1 Table — (DOCX) [file pone.0268421.s001.docx]

**S1 Table. Associations between baseline variables and RRT-free survival amongst those who were given immunosuppression (n=82) using univariate and multivariate Cox regression analysis.**

|  | Univariate model | | Multivariate model | |
| --- | --- | --- | --- | --- |
| Variable | Hazard Ratio (95% CI) | P-value | Hazard Ratio (95% CI) | P-value |
| Age | 1.02 (1.00-1.04) | **0.041** | 0.98 (0.96- 1.01) | 0.160 |
| Male gender | 2.30 (1.05-5.05) | **0.037** | 1.91 (0.78- 4.69) | 0.160 |
| Caucasian | 0.35 (0.08-1.46) | 0.149 | - | - |
| Diabetes | 1.41 (0.77-2.55) | 0.307 | - | - |
| HTN | 2.28 (1.04-5.01) | **0.039** | 1.09 (0.42- 2.88) | 0.855 |
| CVD | 2.26 (0.87-5.83) | 0.093 | - | - |
| SBP | 1.02 (1.00-1.04) | **0.043** | 1.01 (0.99- 1.03) | 0.491 |
| DBP | 1.00 (0.97-1.03) | 0.889 | - | - |
| M score | 0.80 (0.42-1.52) | 0.493 | - | - |
| E score | 1.18 (0.49-2.87) | 0.715 | - | - |
| S score | 1.00 (0.53-1.92) | 0.993 | - | - |
| T score | 1.85 (1.25-2.74) | **0.002** | 1.13 (0.71-1.81) | 0.617 |
| C score | 1.08 (0.69-1.70) | 0.732 | - | - |
| Total MEST score | 1.25 (0.97-1.61) | 0.081 | - | - |
| eGFR, ml/min/1.73m^2^ | 0.97 (0.96-0.99) | **<0.001** | 0.98 (0.96- 0.99) | **0.004** |
| uPCR, mg/mmol | 1.00 (1.00-1.00) | 0.221 | - | - |
| ACEi/ARB | 0.25 (0.13-0.49) | **<0.001** | 0.40 (0.17-0.95) | **0.037** |

Multivariate model adjusted for age, male gender, hypertension, systolic blood pressure, T-score, eGFR and ACEi/ ARB use.

ACEi, angiotensin converting enzyme inhibitor; ARB, angiotensin receptor blocker; C, crescent; CVD, cardiovascular disease; DBP, diastolic blood pressure; E, endocapillary hypercellularity; eGFR, estimated glomerular filtration rate; HTN, hypertension; M, mesangial hypercellularity; S, segmental sclerosis; SBP, systolic blood pressure; T, tubular atrophy and interstitial fibrosis; uPCR, urine protein creatinine ratio.
