# Supplementary material for: The epidemiology and evolution of IgA nephropathy over two decades: A single centre experience
Source: PLoS One. 2022 Sep 1;17(9):e0268421. doi: 10.1371/journal.pone.0268421 (PMC9436111; doi:10.1371/journal.pone.0268421)
Supplement: S2 Table — (DOCX) [file pone.0268421.s002.docx]

**S2 Table. Baseline characteristics, laboratory values and outcomes of cohort by rate of eGFR decline.**

|  | Delta eGFR <-1ml/min/1.73m^2^/year (n=159) | Delta eGFR -1 to -5ml/min/1.73m^2^ (n=93) | Delta eGFR > -5ml/min/1.73m^2^ (n=94) | P value (comparing delta eGFR >-5 with delta eGFR <-1) |
| --- | --- | --- | --- | --- |
| Age, years | 44.0 (29.0-63.0) | 46.0 (31.0-57.5) | 42.6 (29.0-67.5) | 0.372 |
| Male | 108 (67.9) | 69 (74.2) | 64 (68.1) | 0.538 |
| Caucasian | 147 (92.5) | 79 (84.9) | 78 (83) | 0.078 |
| Diabetes | 10 (6.3) | 6 (6.5) | 8 (8.5) | 0.78 |
| Hypertension | 84 (52.8) | 63 (67.7) | 58 (61.7) | 0.057 |
| CVD | 13 (8.2) | 6 (6.5) | 10 (10.6) | 0.582 |
| SBP, mmHg | 120 (110-132) | 120 (110-130) | 126 (117-135) | 0.518 |
| DBP, mmHg | 79 (70-85) | 79 (70-90) | 82 (75-89.5) | **0.003** |
| IgA, g/L | 4.14 (3.01-5.36) | 4.17 (2.97-5.28) | 3.33 (2.74-4.50) | **0.011** |
| C3, g/L | 1.22 (1.03-1.42) | 1.22 (1.01-1.41) | 1.27 (1.00-1.46) | 0.733 |
| Haemoglobin, g/L | 126 (109.25-143.0) | 129 (115-141) | 124 (114.25-138.75) | 0.676 |
| Albumin, g/L | 39 (34-43) | 40 (34-43) | 39 (36-43) | 0.453 |
| ALP, U/L | 70 (58.25-89.75) | 70 (61.75-83.0) | 71 (60-90) | 0.818 |
| P04, mmol/L | 1.19 (1.03- 1.34) | 1.18 (1.00-1.36) | 1.20 (1.00-1.40) | 0.799 |
| CCa, mmol/L | 2.28 (2.17-2.34) | 2.30 (2.20-2.39) | 2.25 (2.10-2.33) | 0.143 |
| Creatinine at biopsy, µmol/L | 117 (87.5-213) | 161 (109.25-242.25) | 142 (86-230.25) | 0.636 |
| eGFR at biopsy, ml/min/1.73m2 at biopsy | 57.2 (27.6-84.5) | 42.1 (24.8-63.7) | 51.0 (28.8-88.5) | 0.808 |
| uPCR at biopsy, mg/mmol | 122.50 (48.5-341) | 184 (92-319) | 230 (124.25-413.25) | **0.001** |
| ACEi/ ARB | 133 (84.2) | 76 (81.7) | 83 (88.3) | 0.449 |
| Immunosuppression | 32 (20.1) | 18 (19.4) | 19 (20.2) | 0.986 |
| RRT | 10 (6.3) | 36 (38.7) | 48 (51.1) | **<0.001** |
| Mortality | 27 (17.0) | 14 (15.1) | 16 (17.0) | 0.911 |
| Follow up duration, months | 77 (33-117) | 77 (42.5-121.5) | 30.5 (18-61.75) | **<0.001** |

Continuous variables are presented as median (interquartile range), p-value by Mann–Whitney U-test. Categorical variables presented as number (percentage), p-value by Chi-squared test.

Delta eGFR < -1ml/min/1.73m^2/^year = Fall in eGFR of less than 1ml/min/1.73m^2^/ year

Delta eGFR -1 to -5ml/min/1.73m^2^/year= Fall in eGFR of between 1 and 5ml/min/1.73m^2^/year

Delta eGFR > -5ml/min/1.73m^2^/year= Fall in eGFR of more than 5ml/min/1.73m^2^/year

ACEi, angiotensin converting enzyme inhibitor; ALP, alkaline phosphatase; ARB, angiotensin receptor blockade; C3, complement 3; CCa, corrected calcium; CVD, cardiovascular disease; DBP, diastolic blood pressure; eGFR, estimated glomerular filtration rate; IgA, immunoglobulin; P04, phosphate; RRT, renal replacement therapy; SBP, systolic blood pressure; uPCR, urine protein creatinine ratio.

n=346, the number of subjects who had a delta eGFR result available.
