# Supplementary material for: The epidemiology and evolution of IgA nephropathy over two decades: A single centre experience
Source: PLoS One. 2022 Sep 1;17(9):e0268421. doi: 10.1371/journal.pone.0268421 (PMC9436111; doi:10.1371/journal.pone.0268421)
Supplement: S3 Table — (DOCX) [file pone.0268421.s003.docx]

**Supplementary Table 3. Baseline characteristics and outcomes according to timing of biopsy- 2000-2011 vs 2012 onwards.**

| Variable | 2000-2011 (n=215) | 2012-2019 (n=186) | P-value |
| --- | --- | --- | --- |
| Age, years | 48 (31-63) | 43 (29-60) | 0.171 |
| Male | 159 (74.0) | 120 (64.5) | **0.041** |
| Caucasian | 192 (89.3) | 159 (85.5) | 0.413 |
| Diabetes | 14 (6.5) | 16 (8.6) | 0.427 |
| Hypertension | 129 (60.0) | 102 (54.8) | 0.297 |
| CVD | 19 (8.8) | 18 (9.7) | 0.772 |
| SBP, mmHg | 132 (123-143) | 130 (120-143) | 0.215 |
| DBP, mmHg | 80 (70-86) | 80 (70-88.5) | 0.439 |
| IgA, g/L | 4.07 (2.93-5.34) | 3.79 (2.98- 4.92) | 0.662 |
| C3, g/L | 1.21 (1.00-1.39) | 1.20 (1.00-1.46) | 0.582 |
| Haemoglobin, g/L | 124 (104-142) | 124.5 (110-141) | 0.524 |
| Albumin, g/L | 39 (34-43) | 40 (34-43) | 0.885 |
| ALP, U/L | 70 (60-86) | 71 (59.5-92) | 0.560 |
| P04, mmol/L | 1.22 (1.05-1.40) | 1.17 (1.00-1.50) | 0.343 |
| CCa, mmol/L | 2.31 (2.17-2.41) | 2.26 (2.10-1.32) | **0.012** |
| Creatinine at biopsy, µmol/L | 144 (92.8-245.3) | 139 (90.3-230.5) | 0.240 |
| eGFR at biopsy, ml/min/1.73m2 | 45.5 (24.3-82.2) | 48.3 (25.2-82.6) | 0.327 |
| uPCR, mg/mmol | 165 (62.0-377.8) | 195 (94.5-431) | 0.091 |
| ACEi/ ARB | 177 (82.3) | 142 (76.3) | 0.092 |
| Immunosuppression | 35 (16.3) | 47 (25.3) | **0.026** |
| RRT | 83 (38.6) | 30 (16.1) | **<0.001** |
| Mortality | 60 (27.9) | 19 (10.2) | **<0.001** |
| Follow up duration, months | 75 (26-132) | 34.5 (14.75-66.5) | **<0.001** |

Continuous variables are presented as median (interquartile range), p-value by Mann–Whitney U-test. Categorical variables presented as number (percentage), p-value by Chi-squared test.

ACEi, angiotensin converting enzyme inhibitor; ALP, alkaline phosphatase; ARB, angiotensin receptor blockade; C3, complement 3; CCa, corrected calcium; CVD, cardiovascular disease; DBP, diastolic blood pressure; eGFR, estimated glomerular filtration rate; IgA, immunoglobulin; P04, phosphate; RRT, renal replacement therapy; SBP, systolic blood pressure; uPCR, urine protein creatinine ratio.
