# Supplementary figures and images for: The epidemiology and evolution of IgA nephropathy over two decades: A single centre experience
Source: PLoS One. 2022 Sep 1;17(9):e0268421. doi: 10.1371/journal.pone.0268421 (PMC9436111; doi:10.1371/journal.pone.0268421)

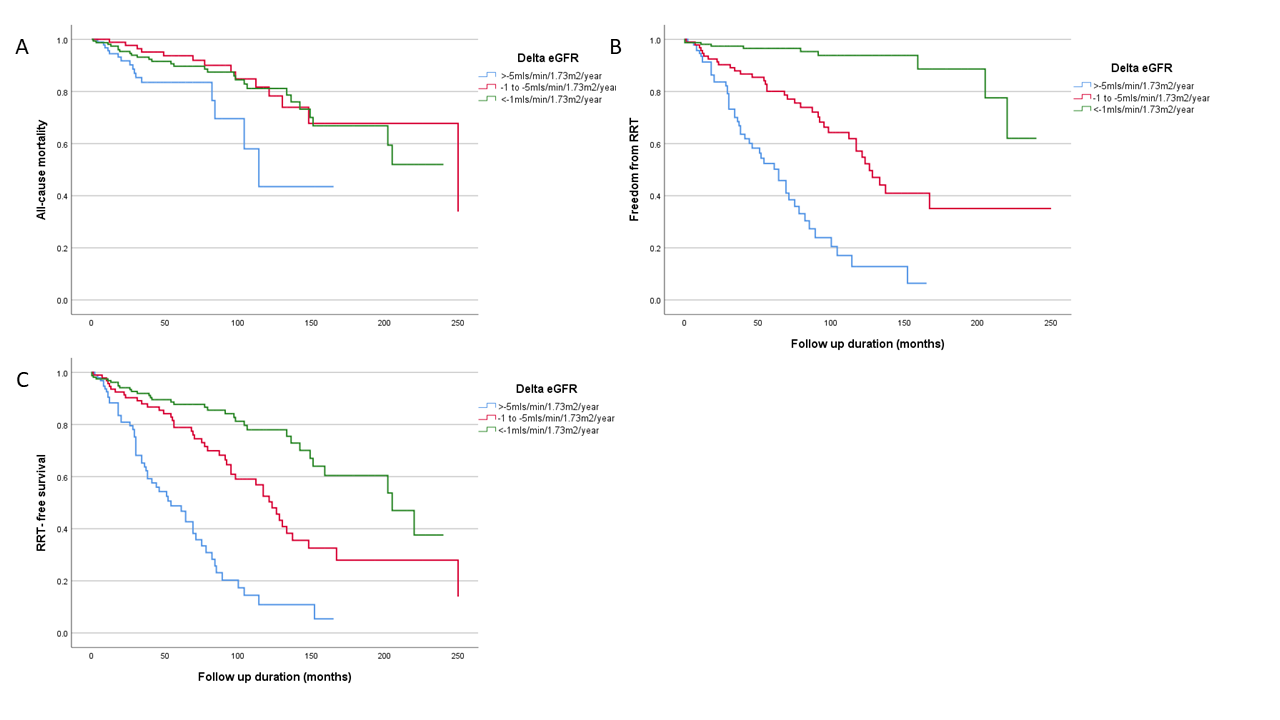

Supplement: S1 Fig — Kaplan-Meier curves for all-cause mortality (A), freedom from RRT (B) and RRT-free survival (C) by rate of eGFR decline (>-5ml/min, -1 to -5ml/min and <-1ml/min). P-values 0.012, <0.001 and <0.001 respectively. (TIF) [file pone.0268421.s004.tif]

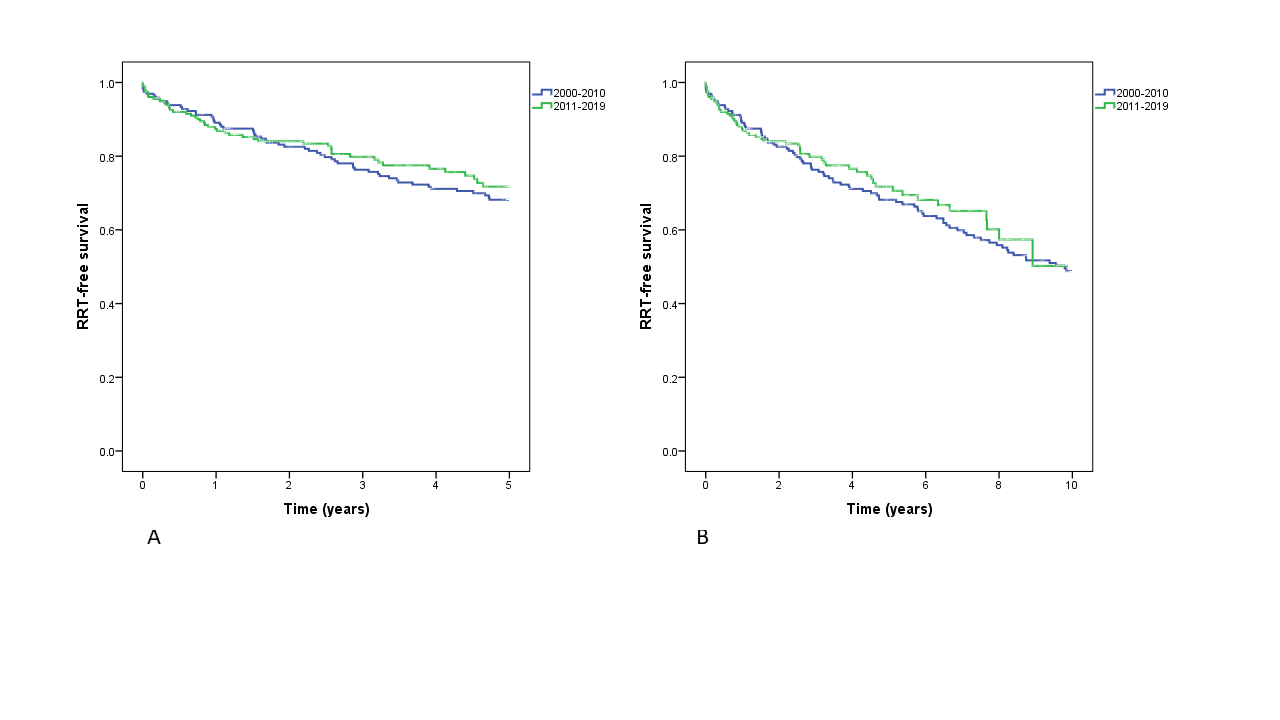

Supplement: S2 Fig — Kaplan-Meier curves for 5 year (A) and 10 year (B) RRT-free survival by timing of biopsy (2000–2010 vs 2011–2019). (TIF) [file pone.0268421.s005.tif]

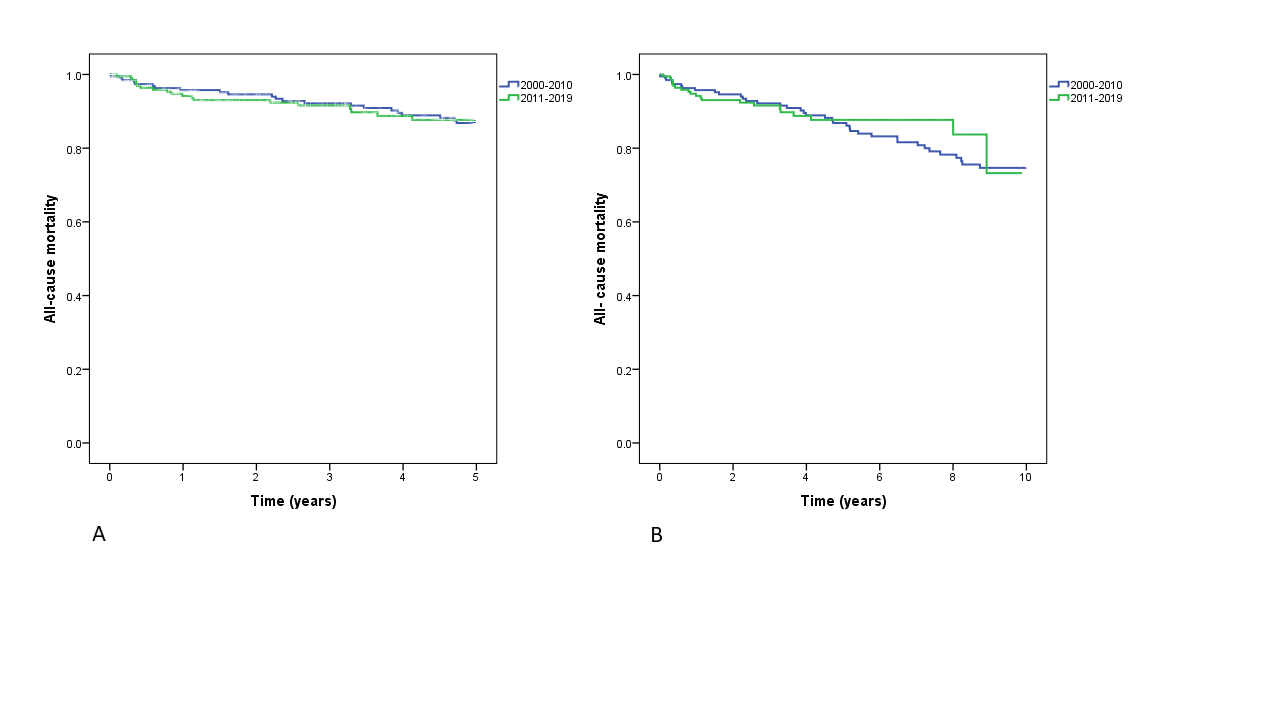

Supplement: S3 Fig — Kaplan-Meier curves for 5 year (A) and 10 year (B) all-cause mortality by timing of biopsy (2000–2010 vs 2011–2019). (TIF) [file pone.0268421.s006.tif]
