# Supplementary material for: The epidemiology and evolution of IgA nephropathy over two decades: A single centre experience
Source: PLoS One. 2022 Sep 1;17(9):e0268421. doi: 10.1371/journal.pone.0268421 (PMC9436111; doi:10.1371/journal.pone.0268421)
Supplement: S1 File — (DOCX) [file pone.0268421.s007.docx]

**Effect of eGFR decline**

The overall cohort was also subdivided according to the rate of eGFR decline (supplementary table 1). The cohort was split into 3 groups- those with eGFR decline better than -1ml/min/1.73m^2^/year (n=159; group A), those with eGFR decline between -1 and -5ml/min/1.73m^2^/year (n=93; group B), and those with eGFR decline worse than -5ml/min/1.73m^2^/year (n=94; group C). The groups were generally well matched, with the main differences as the rates of decline increased being i) a higher diastolic blood pressure (79mmHg, 79mmHg and 82mmHg for groups A, B and C, respectively), ii) a lower serum IgA level (4.14g/L, 4.17g/L and 3.33 g/L respectively), iii) an upward trend in uPCR (112.5mg/mmol, 185mg/mmol and 230mg/mmol respectively, and unsurprisingly greater requirement for RRT (6.3%, 38.7% and 51.1% for respectively). There were no major differences in immunosuppression use and mortality between the 3 groups. The follow up duration was significantly less in the fastest declining group when compared to the slowest declining group (30.5 vs 77 months; p<0.001). Supplementary fig 1 shows Kaplan-Meier curves for all-cause mortality (A), freedom from RRT (B) and RRT-free survival (C) with clear separation between the three groups.
